# Exposure to Volatile Organic Compounds in Relation to Visceral Adiposity Index and Lipid Accumulation Product Among U.S. Adults: NHANES 2011–2018

**DOI:** 10.3390/toxics13010046

**Published:** 2025-01-09

**Authors:** Ziyi Qian, Chenxu Dai, Siyan Chen, Linjie Yang, Xia Huo

**Affiliations:** 1 Laboratory of Environmental Medicine and Developmental Toxicology, Guangdong Key Laboratory of Environmental Pollution and Health, College of Environment and Climate, Jinan University, Guangzhou 511443, China; zyqian62@163.com (Z.Q.); dcxdyx@stu2022.jnu.edu.cn (C.D.); sychen@stu2022.jnu.edu.cn (S.C.); ylj15625586427@163.com (L.Y.); 2 Department of Public Health and Preventive Medicine, School of Medicine, Jinan University, Guangzhou 510632, China

**Keywords:** volatile organic compounds, urinary metabolites, visceral adiposity index, lipid accumulation product, National Health and Nutrition Examination Survey

## Abstract

Volatile organic compounds (VOCs) are associated with obesity health risks, while the association of mixed VOCs with visceral adiposity indicators remains unclear. In this study, a total of 2015 adults from the National Health and Nutrition Examination Survey (NHANES) were included. Weighted generalized linear models, restricted cubic spline (RCS), weighted quantile sum (WQS), and Bayesian kernel machine regression (BKMR) were adopted to assess the association of VOC metabolites (mVOCs) with the visceral adiposity index (VAI) and lipid accumulation product (LAP). Multiple mVOCs were positively associated with the VAI and LAP in the single-exposure model, especially N-acetyl-S-(2-carboxyethyl)-L-cysteine (CEMA) and N-acetyl-S-(N-methylcarbamoyl)-L-cysteine (AMCC). The associations of mVOCs with VAI and LAP were more significant in <60-year-old and non-obese individuals, with interactions of CEMA with age and AMCC with obesity status. Nonlinear relationships between certain mVOCs and the VAI or the LAP were also observed. In the WQS model, co-exposure to mVOCs was positively correlated with the VAI [β (95%CI): 0.084 (0.022, 0.147)]; CEMA (25.24%) was the major contributor. The result of the BKMR revealed a positive trend of the association between mixed mVOCs and the VAI. Our findings suggest that VOC exposure is strongly associated with visceral obesity indicators. Further large prospective investigations are necessary to support our findings.

## 1. Introduction

Obesity is defined as an excessive buildup of body fat. According to the World Health Organization (WHO), more than 890 million adults worldwide were obese (16%) as of 2022 [1]. Obesity is associated with the prevalence of many diseases, notably metabolic diseases such as type 2 diabetes, cardiovascular disease, and hypertension [2,3,4]. It is important to note that individuals with a comparable body mass index (*BMI*) vary in their body fat distribution, metabolic profile, and the degree of related metabolic risk [5]. Obesity health risks are primarily affected by body fat distribution, with visceral fat (rather than subcutaneous fat) being a more crucial risk factor [5,6,7]. A previous review referred to metabolic syndrome as “visceral adiposity syndrome”. The expansion of visceral adipose tissue stimulates an inflammatory response and produces cytokines that directly interfere with insulin signaling, leading to insulin resistance, which is a key risk factor for metabolic disorders [8]. Genetics and lifestyle (e.g., diet and physical activity) have a remarkable effect on obesity health risk. Moreover, studies suggest that environmental pollutants are also related to insulin resistance, glucose and lipid homeostasis, and obesity [9,10].

Volatile organic compounds (VOCs) are ubiquitous pollutants, originating primarily from the emissions of numerous day-to-day products used in residential and commercial applications [11]. VOCs are predominantly found in the air, especially indoors, where multiple sources of exposure exist alongside inadequate ventilation [12]. This results in a wider and easier VOC exposure for the general population. Previous studies suggested that VOC exposure has adverse effects on human health and is correlated with the risk of several diseases, such as respiratory diseases, cancers, type 2 diabetes, cardiovascular diseases, and metabolic syndrome [11,13,14,15,16]. A recent cross-sectional study showed that concentrations of multiple VOC metabolites (mVOCs) were associated with obesity or abdominal obesity in adults [17]. However, conventional measurements of obesity, such as *BMI* and waist circumference (*WC*), only provide a crude assessment of obesity and cannot distinguish between subcutaneous and visceral fat. Epidemiological data on the association between VOC exposure (especially mixed exposure) and visceral adiposity are still sparse.

Various measurements of obesity have been developed and used in epidemiologic investigations. Some simple, inexpensive, and effective identifiers of visceral obesity have attracted attention, such as the visceral adiposity index (*VAI*) and lipid accumulation product (*LAP*). They involve the integration of anthropometric and lipid metabolic parameter information to effectively identify visceral obesity and associated cardiometabolic risk [5]. The *VAI* is useful for assessing visceral fat distribution and dysfunction [18]. The *LAP* serves to assess abdominal lipid accumulation status [19,20]. The identification of potential VOCs related to the *VAI* or *LAP* can contribute to a deeper understanding of the mechanisms by which VOCs affect obesity and its related health effects.

In this study, we aimed to systematically investigate the associations between VOC exposure and visceral adiposity indicators (i.e., the *VAI* and *LAP*) in general adults using data from the National Health and Nutrition Examination Survey (NHANES). Upon exposure, VOCs undergo metabolic transformation and are excreted as one or more mercapturic acids [21]. Considering that the physiological half-life of urinary mVOCs in urine is longer than that of parent VOCs in blood and considering the specificity of most mercapturic acid metabolites, urinary mVOCs were used as the indicator of VOC exposure in this study [21,22,23]. Our findings will provide new evidence to further elucidate the complex associations between chemical exposure and obesity.

## 2. Methods

### 2.1. Study Population

The NHANES is a large-scale, cross-sectional survey conducted in 2-year cycles to assess the nutritional and health status of the U.S. general population. Participants all provided informed consent to participate in the program. The population for this study was drawn from four NHANES cycles (2011–2012, 2013–2014, 2015–2016, and 2017–2018). Survey data were collected through household interviews, standardized body measures, and laboratory tests [24]. Our study focused on adults (≥20 years of age, *n* = 22617). Pregnant individuals (*n* = 247) were excluded. Figure S1 shows the flowchart of participant screening. Finally, this study included 2015 participants.

### 2.2. Measurement of Urinary VOC Metabolites

Urinary mVOCs were quantified using ultra-performance liquid chromatography–electrospray tandem mass spectrometry (UPLC-ESI/MSMS) as outlined by Alwis et al. [22]. Detailed information and experimental protocols are available in the laboratory method file (https://wwwn.cdc.gov/nchs/data/nhanes/public/2013/labmethods/UVOC_H_MET.pdf) (accessed on 26 December 2024).

The lower limits of detection (LLODs) for the metabolites remained consistent across all four survey cycles. Analyte concentrations below the LLOD were assigned a value of LLOD divided by the square root of 2. We excluded analytes with less than 75% detection and ultimately selected 16 mVOCs for analysis: N-acetyl-S-(2-carboxyethyl)-L-cysteine (CEMA), N-acetyl-S-(3-hydroxypropyl-1-methyl)-L-cysteine (HMPMA), N-acetyl-S-(4-hydroxy-2-butenyl)-L-cysteine (MHBMA3), N-acetyl-S-(2-cyanoethyl)-L-cysteine (CYMA), N-acetyl-S-(N-methylcarbamoyl)-L-cysteine (AMCC), N-acetyl-S-(2-hydroxypropyl)-L-cysteine (2HPMA), 2-methylhippuric acid (2MHA), N-acetyl-S-(3-hydroxypropyl)-L-cysteine (3HPMA), 3- and 4-methylhippuric acid (3-4MHA), N-acetyl-S-(2-carbamoylethyl)-L-cysteine (AAMA), 2-aminothiazoline-4-carboxylic acid (ATCA), N-acetyl-S-(n-propyl)-L-cysteine (BPMA), N-acetyl-S-(3,4-dihydroxybutyl)-L-cysteine (DHBMA), mandelic acid (MA), N-acetyl-S-(benzyl)-L-cysteine (BMA), and phenylglyoxylic acid (PGA). The parent compounds, LLODs, detection rates, and concentration distributions of these metabolites are presented in Table S1. Urine creatinine was introduced to correct mVOC levels to account for urine dilution. Specifically, mVOC levels were corrected by dividing them by the creatinine concentrations and ultimately expressed in ug/g creatinine (ug/g Cr).

### 2.3. Assessment of Outcomes

Serum samples for triglycerides (*TGs*) and high-density lipoprotein cholesterol (*HDL-C*) testing were collected after 8–12 h of fasting. Measurements of *WC* and *BMI* were performed by trained technicians in the Mobile Examination Center. The *VAI* and *LAP* were calculated according to the equations established in previous studies [18,20]. The equations are as follows:

(1) $$Males\text{:}\;VAI=\left(\frac{WC}{39.68+1.88\times BMI}\right)\times \left(\frac{TG}{1.03}\right)\times \left(\frac{1.31}{HDL-C}\right)$$

(2) $$Females\text{:}\;VAI=\left(\frac{WC}{36.58+1.89\times BMI}\right)\times \left(\frac{TG}{0.81}\right)\times \left(\frac{1.52}{HDL-C}\right)$$

(3) $$Males\text{:}\;LAP=\left(WC-65\right)\times TG$$

(4) $$Females\text{:}\;LAP=\left(WC-58\right)\times TG$$

where *WC* and *BMI* are in cm and kg/m^2^, respectively, and *TGs* and *HDL-C* are in mmol/L.

### 2.4. Assessment of Covariates

Covariates were calculated or categorized based on previous studies, including demographic variables, lifestyle, and chronic diseases [23,25,26,27,28,29]. Specifically included were sex, age, race, education level, poverty-to-income ratio (PIR), *BMI*, self-reported smoking status, serum cotinine, average daily alcohol consumption, physical activity, Healthy Eating Index-2015 (HEI-2015), diabetes, hypertension, and cardiovascular disease (CVD). The HEI-2015 was calculated using the R package “dietaryindex” [30], based on the data of total nutrient intakes from the first 24 h dietary recall interview (data file “DR1TOT”). Additional details on covariates are provided in Text S1 of the Supplementary Materials.

### 2.5. Statistical Analysis

Descriptive statistics were performed to analyze the baseline characteristics of the population. Categorical variables were reported as counts (percentages, %) and compared using chi-square tests. Continuous variables were reported as mean (standard deviation, SD) or medians [interquartile range, IQR] and were compared using parametric or nonparametric tests. Due to the right-skewed distribution, the *VAI*, *LAP*, and mVOC concentrations were natural log transformed (ln-transformed) before formal analysis. A Pearson correlation analysis was used to examine correlations among the mVOCs. Given the complex survey design of NHANES, for weighted analyses, we used the strata, cluster, and environmental subsample weight data.

#### 2.5.1. Individual VOC Metabolite Exposure Analysis

Associations of individual mVOCs with the *VAI* or *LAP* were examined using survey-weighted generalized linear models (WGLMs). The regression coefficient (β) was interpreted as the average change in the ln-transformed *VAI* or *LAP* for each unit increase in each ln-transformed mVOC. Moreover, restricted cubic spline (RCS) models were fitted as sensitivity analyses. RCS models can capture both linear and nonlinear relationships between exposures and outcomes. The number of RCS knots for each mVOC was determined based on the minimum value of the Akaike information criterion to reduce the possibility of RCS under- and over-fitting. We also stratified the analyses by sex (males or females), age (<60 or ≥60 years of age), and obesity status (*BMI* < 30 kg/m^2^ is considered non-obesity, ≥30 kg/m^2^ is considered obesity). Interaction effects were analyzed by introducing interaction terms into the models.

#### 2.5.2. Mixed VOC Metabolite Exposure Analysis

We used two mixed-exposure models to assess the relationship between mixed mVOCs and the *VAI* or *LAP*. The weighted quantile sum (WQS) regression analysis assesses the overall effect of mixed mVOCs by constructing weighted indices [31]. Parameter estimation and significance tests for the WQS index effect of the mixture were conducted to determine the association between the index and the outcome. The estimated weights of each mVOC reflect its contribution to the overall effect, with weights exceeding the threshold indicating greater contributions. The threshold is typically the inverse of the number of elements in the mixture. Given the limitations of the WQS on detecting effect direction, both positive and negative models were applied. Bayesian kernel machine regression (BKMR) models were further fitted to assess the exposure–response relationship between exposure and outcome as well as the overall effect of mixed exposures [32]. Specifically, single-exposure response function curves reflect the univariate relationship between each mVOC and outcome when all other mVOCs are fixed at the median. The overall effect of mVOCs was assessed by comparing mixtures of mVOCs fixed at specific percentiles (e.g., 25th and 75th) with those fixed at the median. Conditional posterior inclusion probability (PIP) was used to determine the importance of mVOCs on outcome, with the threshold set at 0.5 [33].

All analyses and plots were performed using R (version 4.3.2) and GraphPad Prism (version 10.1.0) software. Statistical significance was defined as a two-tailed *p*-value < 0.05. Weighted generalized linear models, RCS, WQS, and BKMR analyses were conducted using the R packages “survey”, “plotRCS”, “gWQS”, and “bkmr”, respectively.

## 3. Results

### 3.1. Baseline Characteristics of the Study Population

Table 1 demonstrates the survey-weighted baseline characteristics of the 2015 participants in this study. Participants were grouped by sex and included 1057 males and 958 females. The weighted mean (SD) age for all participants was 47.51 (16.72). The weighted medians [IQR] for the *VAI* and *LAP* were 1.33 [0.78, 2.31] and 42.22 [21.88, 72.13], respectively. Non-Hispanic whites (weighted percentage: 69.26%), above high school (62.85%), and *BMI* ≥ 30 kg/m^2^ (39.29%) accounted for a higher percentage of their subgroups. Comparative analyses revealed statistically significant differences between males and females in age, race, *BMI*, smoking, cotinine exposure, alcohol consumption, HEI-2015, physical activity, and CVD prevalence. We also examined demographic information stratified by *VAI*- or *LAP*-weighted tertiles (Tables S2 and S3). The results showed that the three *VAI* or *LAP* groups differed significantly in age, PIR, race, education, *BMI*, alcohol consumption, HEI-2015, and physical activity. The prevalence of hypertension, diabetes, and CVD increased with increasing tertiles of the *VAI* or the *LAP*.

### 3.2. Distribution and Correlation of Urinary VOC Metabolites

**Figure 1:**
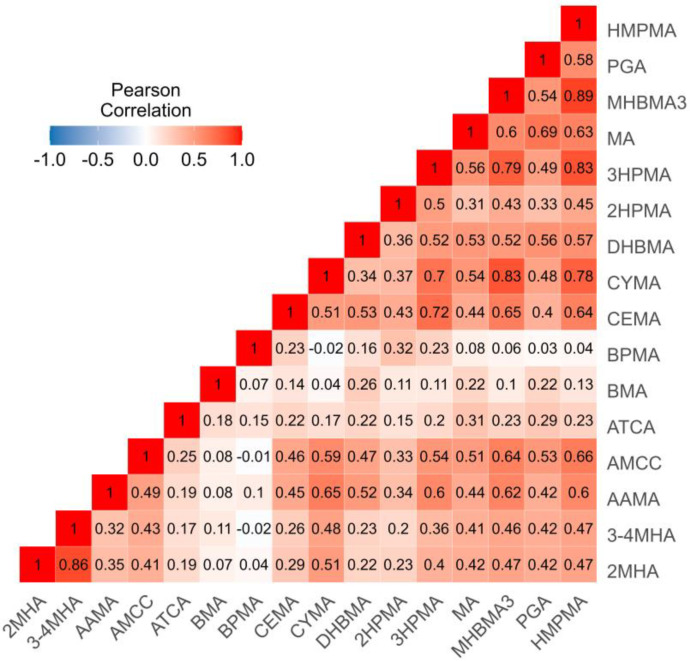
Correlation matrix for urinary VOC metabolites (ln-transformed, μg/g Cr). Notes: CEMA, N-acetyl-S-(2-carboxyethyl)-L-cysteine; HMPMA, N-acetyl-S-(3-hydroxypropyl-1-methyl)-L-cysteine; MHBMA3, N-acetyl-S-(4-hydroxy-2-butenyl)-L-cysteine; CYMA, N-acetyl-S-(2-cyanoethyl)-L-cysteine; AMCC, N-acetyl-S-(N-methylcarbamoyl)-L-cysteine; 2HPMA, N-acetyl-S-(2-hydroxypropyl)-L-cysteine; 2MHA, 2-methylhippuric acid; 3HPMA, N-acetyl-S-(3-hydroxypropyl)-L-cysteine; 3-4MHA, 3- and 4-methylhippuric acid; AAMA, N-acetyl-S-(2-carbamoylethyl)-L-cysteine; ATCA, 2-aminothiazoline-4-carboxylic acid; BPMA, N-acetyl-S-(n-propyl)-L-cysteine; DHBMA, N-acetyl-S-(3,4-dihydroxybutyl)-L-cysteine; MA, mandelic acid; BMA, N-acetyl-S-(benzyl)-L-cysteine; and PGA, phenylglyoxylic acid.

The distribution of creatinine-corrected urinary mVOC concentrations is shown in Table S4. The weighted median level of DHBMA was the highest among the 16 mVOCs, followed by 3-4MHA, PGA, and 3HPMA, and the lowest was CYMA. In addition, several mVOCs (3-4MHA, AMCC, ATCA, BMA, DHBMA, MA, and PGA) were significantly higher in females than in males, while 3HPMA was significantly higher in males. Levels of mVOCs were also characterized according to age and obesity status. Specific information is shown in Tables S5 and S6. Urinary concentrations of mVOCs were bivariate-correlated with the Pearson correlation coefficients from −0.02 to 0.89 (*p* < 0.001 for most correlations) (Figure 1). CYMA, 3HPMA, MHBMA3, and HMPMA were highly correlated with each other (r = 0.70–0.89). There were also high correlations between 2MHA and 3-4MHA (r = 0.86) and between CEMA and 3HPMA (r = 0.72). Other statistically significant correlations were relatively weak or moderate, with correlation coefficients ranging from 0.06–0.69. This suggests that these mVOCs probably have similar sources or relatively strong collinearity.

### 3.3. Association of Individual Urinary VOC Metabolites with VAI and LAP

**Figure 2:**
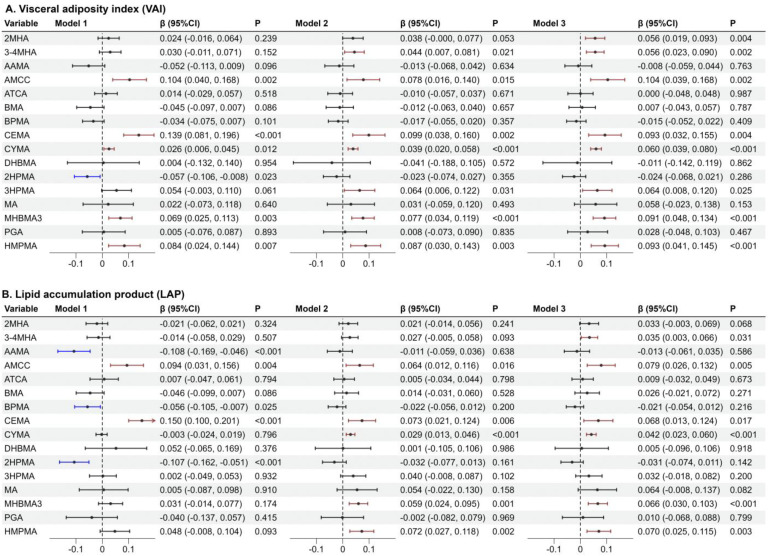
The association analysis between mVOCs and the *VAI* or the *LAP*: (**A**) for the *VAI* and (**B**) for the *LAP*. Model 1 is a crude model unadjusted for any covariates. Model 2 was adjusted for sex, age, race, education, PIR, and *BMI*. Model 3 was further adjusted for serum cotinine, physical activity, HEI-2015, self-reported smoking status, average daily alcohol consumption, diabetes, hypertension, and CVD based on model 2.

Three weighted generalized linear models were fitted to explore univariate associations between individual mVOCs and the *VAI* and *LAP*. In all three models, AMCC, CEMA, CYMA, MHBMA3, and HMPMA were significantly positively associated with the *VAI* (Figure 2A); only AMCC and CEMA were significantly positively associated with *LAP* (Figure 2B). In crude model 1, increased exposure to AAMA, BPMA, and 2HPMA were all significantly associated with a decreased level of the *LAP*, and an increased level of 2HPMA was also significantly associated with a decreased level of the *VAI*. These negative associations were not significant after covariate adjustment. In the fully covariate-adjusted model 3, increased levels of 3-4MHA, AMCC, CEMA, CYMA, MHBMA3, and HMPMA were significantly associated with increased levels of the *VAI* and *LAP*, respectively. In addition, significant positive associations of 2MHA and 3HPMA with the *VAI* were observed in model 3.

Sensitivity analyses further employed RCS models to flexibly fit and visualize linear and nonlinear relationships between mVOCs and the *VAI* or the *LAP*. As shown in Figures S2 and S3, the RCS showed broadly similar results to the weighted generalized linear models. Consistently, we observed that AMCC, CEMA, CYMA, 3HPMA, MHBMA3, and HMPMA were positively correlated with the *VAI* (*p* for overall < 0.05 for all, *p* for nonlinear > 0.05 except for AMCC) and that AMCC, CYMA, and MHBMA3 were positively correlated with the *LAP* (*p* for overall < 0.05, *p* for nonlinear > 0.05). The results also revealed nonlinear correlations of AAMA, ATCA, 2HPMA, and PGA with the *VAI* or the *LAP* (*p* for nonlinear < 0.05), as well as a significant overall negative trend for 2HPMA and PGA (*p* for overall < 0.05).

#### Associations of Urinary VOC Metabolites with *VAI* and *LAP* in Stratified Analyses

**Figure 3:**
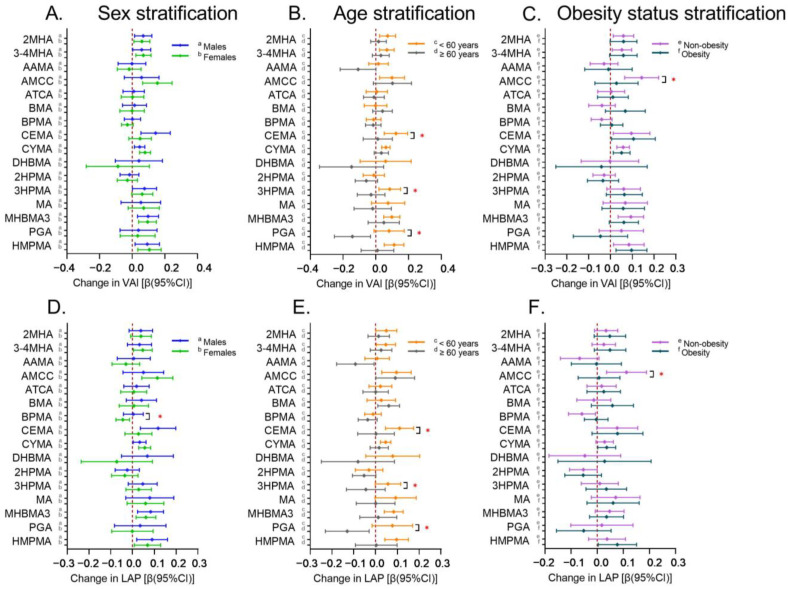
Estimated effects of urinary mVOCs on *VAI* and *LAP* grouped by sex (**A** for *VAI*, **D** for *LAP*), age (**B** for *VAI*, **E** for *LAP*), and obesity status (**C** for *VAI*, **F** for *LAP*) based on interaction models. Notes: *, *p* for interaction < 0.05; obesity status was categorized as non-obesity (*BMI* < 30 kg/m^2^) and obesity (*BMI* ≥ 30 kg/m^2^).

Stratified subgroup analyses of sex, age, and obesity status were conducted to identify populations sensitive to the effects of VOC exposure on the *VAI* or *LAP*. The results are summarized in Figure 3. For the *VAI*, 2MHA, 3-4MHA, CYMA, MHBMA3, and HMPMA were significantly and positively associated with the *VAI* regardless of sex subgroups. CEMA and AMCC were positively associated with the *VAI* in males and females, respectively. After stratification by age and obesity status, the majority of positive associations were observed in the <60 years of age and non-obesity subgroups. In the subgroup of <60 years of age, 2MHA, 3-4MHA, AMCC, CEMA, CYMA, 3HPMA, MHBMA3, and HMPMA were positively associated with the *VAI*, with significant interactions with age for CEMA (*p* for interaction = 0.019) and 3HPMA (*p* for interaction = 0.046). In the subgroup of non-obesity, 2MHA, 3-4MHA, AMCC, CEMA, CYMA, MHBMA3, and HMPMA were positively associated with the *VAI*, and AMCC had a significant interaction with *BMI* (*p* for interaction = 0.010). CEMA, CYMA, MHBMA3, and HMPMA were also associated with the *VAI* in the obesity subgroup. For the *LAP*, similar to the *VAI*, multiple mVOCs were significantly and positively associated with the *LAP* in both the <60 years of age and the non-obesity subgroups. In particular, for both the *VAI* and *LAP*, CEMA and 3HPMA showed significant interactions with age, as did AMCC with *BMI*. In addition, PGA was significantly negatively associated with the *VAI* and *LAP* in the subgroup ≥ 60 years of age, and the interaction with age was significant.

### 3.4. The Overall Effect of Mixed mVOCs on VAI and LAP

**Figure 4:**
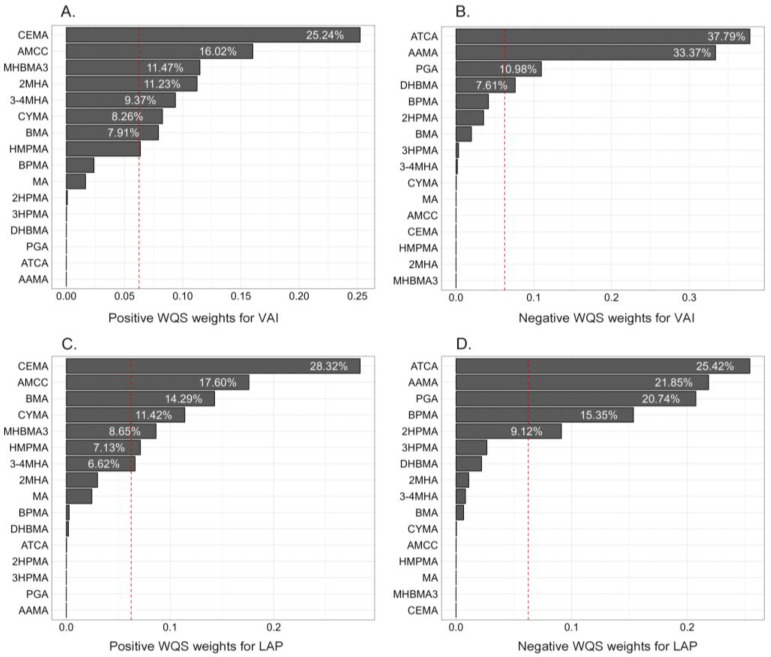
Weights for urinary mVOCs associated with the *VAI* and *LAP* from the weighted quantile sum (WQS) regression model: (**A**) positive weights for the *VAI*, (**B**) negative weights of the *VAI*, (**C**) positive weights for the *LAP*, (**D**) negative weights for the *LAP*. The red dashed line is the weight threshold for determining the importance of an element, which was set as the inverse of the number of elements in the mixture (i.e., 1/16).

Since there are multiple highly correlated mVOCs, the use of traditional multivariate regression methods may result in collinearity and variance inflation problems or may have major limitations in the selection of relevant components. Therefore, we used the WQS and BKMR models to assess the association of mixed exposure to VOCs with the *VAI* and *LAP* and to identify the risk components among mVOCs. Due to the limitations of the WQS model on the direction of association, positive and negative modeling were performed, respectively. After adjusting for covariates, the WQS index for exposure to mixtures of mVOCs was significantly positively correlated with *VAI* levels [β (95% CI): 0.084 (0.022, 0.147), *p* = 0.008], while the positive correlation with the *LAP* was not significant (*p* > 0.05) (Table 2). No significant association of the WQS index with the *VAI* or the *LAP* was observed in the negative WQS regression model. Figure 4 illustrates the WQS weight distribution results. The threshold was set as 1/16. In the positive WQS regression model, for both the *VAI* and *LAP*, CEMA (25.24% for the *VAI* and 28.32% for the *LAP*) and AMCC (16.02% for *VAI* and 17.06% for *LAP*) made the greater contribution to the WQS index.

**Figure 5:**
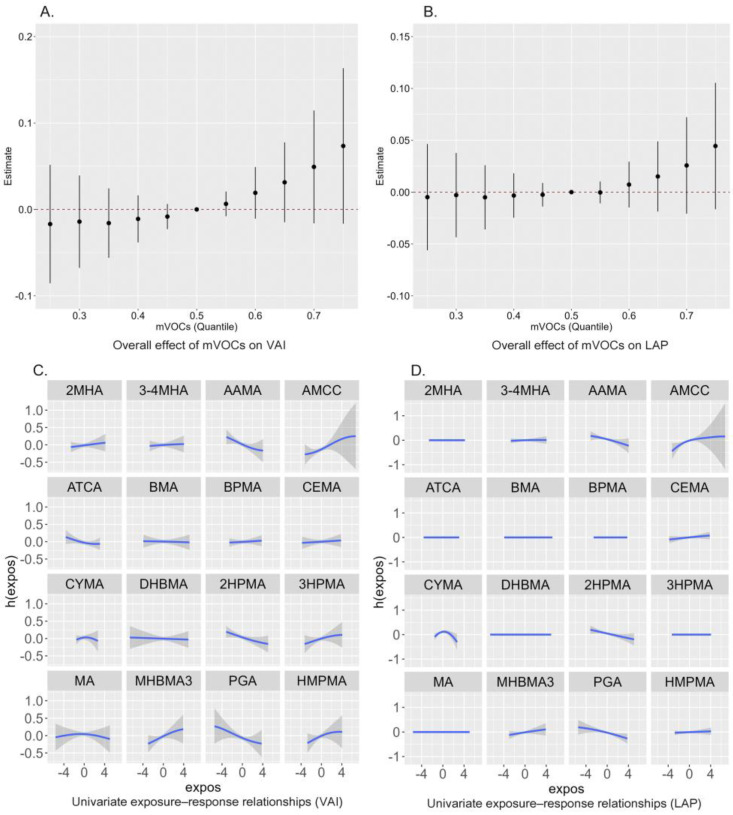
Overall relationship between the mixed mVOCs and the (**A**) *VAI* and (**B**) *LAP* estimated by the Bayesian kernel machine regression (BKMR) model. Univariate exposure–response functions between mVOCs and the (**C**) *VAI* and (**D**) *LAP* estimated by the BKMR model.

The BKMR models with a gaussian kernel function were further fitted to estimate the overall effect of 16 mVOCs. Figure 5A,B demonstrate the estimates of the *VAI* or the *LAP* when all mVOCs are fixed at different percentiles compared to when all are fixed at the 50th percentile. Similar to the results of the WQS model, the result suggested a positive trend of association of mixed mVOCs with the *VAI* and *LAP*, although not significant. According to the results of the univariate exposure–response function, CEMA and AMCC showed a positive trend with both the *VAI* and *LAP* when other mVOCs were fixed at the median (Figure 5C,D). The PIP for mVOCs calculated by the BKMR model was used to quantify the relative importance of mVOC exposure on outcomes, as shown in Table S7. AMCC had the highest PIP values for the *VAI* and *LAP*, indicating that AMCC had the highest contribution to the overall effect.

## 4. Discussion

To the best of our knowledge, a knowledge gap remains regarding the relationship between the exposure to VOCs and visceral obesity. This study explored the potential association between urinary mVOCs and the *VAI* and *LAP*. In the individual exposure analyses, adjusted for different covariates, concentrations of several mVOCs (including AMCC, CEMA, CYMA, MHBMA3, and HMPMA) were consistently associated with increased levels of the *VAI* or the *LAP*. Nonlinear relationships were found among some mVOCs and the *VAI* or the *LAP*. Significant interactions were observed between CEMA and age for the *VAI* and *LAP* and between AMCC and obesity status. In addition, there was also a positive trend of association between mixed mVOCs and the *VAI*.

Several studies suggested that the blood *TG* levels in the presence of large *WC* might be a simple and useful marker representing excess visceral adiposity [34,35]. Although studies directly investigating the link between VOC exposure and visceral adiposity are lacking, previous studies reported the association between VOC exposure and obesity and blood lipid profiles. An observational study found that higher benzene metabolite levels (t,t-MA) were associated with increased obesity and diabetes risk [16]. Similar to our results, Lei et al. reported positive associations of mVOCs (particularly CEMA) with obesity and abdominal obesity in the US general population [17]. Occupational exposure to VOCs in female nail technicians was correlated to elevated *TG* and reduced *HDL-C* levels. Meanwhile, plasma activities of AST and ALT were significantly increased in nail technicians compared to controls, suggesting possible adverse effects of VOC exposure on liver function, which plays a crucial role in lipid metabolism [36,37]. Another study observed significant correlations between certain mVOCs (especially CEMA) and elevated *WC*, *TGs*, fasting glucose, and reduced *HDL-C* [38]. These studies provided part of the support for our findings. Unlike traditional obesity measurement and lipid profiles, the *VAI* and *LAP* have higher accuracy in identifying visceral obesity and are more predictive of multiple metabolic diseases [39,40,41,42,43]. The present study was based on these findings and extended the investigation to visceral adiposity markers, filling an important research gap in this field.

The association between VOC exposure and the *VAI* and *LAP* may be related to insulin resistance. The expansion of visceral adipose tissue leads to inflammation and insulin resistance, which induces or exacerbates the dysregulation of glucose and lipid homeostasis, making individuals more susceptible to metabolic disorders [8,9,44,45]. Environmental pollution interferes with metabolic processes that regulate lipid accumulation and increase body adiposity [46]. Insulin resistance is more strongly related to regional adipose tissue distribution than to total fat mass [47]. Pollutant (e.g., VOCs) exposure can lead to oxidative stress and inflammatory responses and might result in impaired insulin signaling in insulin-sensitive target tissues (including muscle, liver, and adipose tissue), which can in turn trigger insulin resistance [48,49]. This probably interferes with lipid metabolism and further induces the deposition of visceral adiposity. Previous studies suggested that VOC exposure is associated with insulin resistance and impaired glucose and lipid homeostasis [37,50]. In addition, oxidative stress promotes the accumulation of white adipose tissue, stimulation of preadipocyte proliferation and differentiation, and enlargement of mature adipocytes [51]. Notably, VOCs represent a large class of pollutants, each with their unique toxicity and combinatorial toxicity, and possibly affect visceral adiposity through different mechanisms.

The parent compound of CEMA is acrolein, an unsaturated aldehyde found in tobacco smoke, in industrial emissions, and as a byproduct of lipid peroxidation [52]. Studies suggest that acrolein increases cardiovascular metabolic risk by promoting oxidative stress, inflammation, apoptosis, and systemic dyslipidemia [53,54]. Acrolein was also found to inhibit the insulin signaling pathway and reduce glucose uptake by muscle and adipose tissue cells [55,56]. This suggests that acrolein is a risk factor for abnormalities in glucose and lipid metabolism, which might explain the robust positive correlation of CEMA with the *VAI* and *LAP*. As another metabolite of acrolein, 3HPMA also showed a significant positive correlation with the *VAI* in the model adjusted for covariates. The corresponding parent compound of AMCC is N, N-dimethylformamide (DMF), and the liver is the most sensitive target organ for its toxic effects [57]. An animal study showed that DMF exposure in mice can cause heart and liver injuries, and oxidative stress was involved in the toxic effects [58]. Another in vitro study demonstrated that DMF can disrupt liver function by causing oxidative stress, mitochondrial dysfunction, and alterations in key metabolic pathways including N-glycan biosynthesis and bile acid metabolism, leading to lipid deregulation in hepatocytes [59,60]. Hepatic impairment disrupts lipid metabolism, which might lead to fat accumulation. For other mVOCs that showed positive correlations with the *VAI* or the *LAP*, exposure to acrylonitrile (CYMA) or crotonaldehyde (HMPMA) was reported to increase oxidative stress [61,62]. Additionally, 1,3-butadiene (MHBMA3) was found to cause disturbances in metabolism-related enzyme activities, such as the depletion of glutathione and saturation of oxidative metabolism [63]. This induces oxidative stress and the impairment of liver function, probably explaining the positive correlations between these mVOCs and the *VAI* or the *LAP*.

The positive associations of mVOCs with the *VAI* and *LAP* varied across age and obesity status subgroups. Specifically, the associations were more significant in populations <60 years or non-obese. Younger and non-obese individuals may be more sensitive to the effects of mVOCs since they have higher basal metabolic rates, stronger immune responses, and healthier patterns of fat distribution and inflammatory states. Sex-stratified analyses revealed significant associations of CEMA and AMCC with the *VAI* and *LAP* only in males and females, respectively, although no interaction was observed. Acrolein is abundant in cigarette smoke, and Table 1 showed a higher proportion of male smokers and cotinine-exposed individuals compared to females, possibly contributing to the complex exposure profile of males. DMF interferes with the interaction of estrogen receptors with estradiol, potentially making females more sensitive to AMCC [64,65]. Further studies are needed to explore the specific mechanisms by which VOC exposure affects different populations and consider developing targeted prevention and intervention strategies.

Mixed-pollutant exposure is a more realistic environmental health concern compared to single exposure. WQS analyses revealed that exposure to mixed mVOCs was significantly and positively correlated with the *VAI*, with CEMA and AMCC being the most weighted contributors. It was generally consistent with the individual exposure analyses. A trend of positive association of mixed exposure with the *VAI* and *LAP* was also observed in the BKMR model. Notably, the BKMR model showed negative associations of AAMA (acrylamide), ATCA (cyanide), 2HPMA (propylene oxide), and PGA (ethylbenzene and styrene) with the *VAI* or the *LAP*, and similar associations were also shown in the RCS model. Acrylamide and cyanide primarily affect the nervous system, potentially impairing central and peripheral nerve function [66,67]. This probably disrupts appetite regulation and stress response, indirectly affecting fat metabolism. High-dose cyanide exposure is lethal, while lower doses might cause weight loss [67]. This partly explains our results. In addition, several of the above VOCs were reported to have hematological effects or carcinogenicity [68]. For instance, occupational exposure to ethylbenzene can decrease hemoglobin levels, probably leading to anemia [69]. These disorders may be accompanied by malnutrition and increased energy expenditure, reducing body adiposity (including visceral adiposity) content. Further experimental validation is needed to elucidate the specific mechanisms.

This study filled a knowledge gap regarding the association between visceral adiposity and VOC exposure. Multiple mVOCs were found to be associated with an increased *VAI* and *LAP*, which provides new inspiration for obesity health risk prevention. The results of this study were derived from a large nationally representative sample. We adopted multiple statistical models to assess the individual and combined effects of mVOCs on the *VAI* and *LAP* to ensure the robustness of the results. This study also has several limitations. Firstly, a causal relationship between VOC exposure and visceral adiposity could not be determined due to the cross-sectional study design. On the one hand, some common VOCs (e.g., saturated and unsaturated hydrocarbons, aromatic hydrocarbons, and aldehydes) are lipophilic [70]. A high fat content might theoretically lead to a greater accumulation of VOCs in the body, which in turn could lead to higher levels of exposure in obese individuals. However, due to the short half-life of VOCs in the body [22], it is speculated that their accumulation in the body may not be obvious. On the other hand, the accumulation of visceral fat promotes reactive oxygen species (ROS) release and oxidative stress, and certain VOCs can be generated endogenously through the interaction of ROS with underlying cellular components [71]. Therefore, there may be reverse causality between certain VOCs and the *VAI* and *LAP*, and further studies are needed to clarify the corresponding endogenous VOC biomarkers in obese individuals and to explore the mechanisms of VOCs in fat accumulation. Secondly, although the effects of a wide range of covariates have been considered, there may still be residual confounders that affect the results. In addition, there is a lack of studies investigating the mechanisms by which VOC exposure affects fat distribution in animals or humans, and the specific mechanisms are still unclear.

## 5. Conclusions

Our findings revealed that multiple mVOCs are significantly positively associated with levels of visceral adiposity markers (the *VAI* and *LAP*), especially CEMA and AMCC. These associations were more significant in people <60 years old and in non-obese individuals. Exposure to mixed mVOCs also showed a positive association with increased *VAI* levels. In short, this study suggests an association between VOC exposure and visceral obesity. Although the exact mechanism is unclear, we hypothesize that insulin resistance may partially explain these relationships. Prospective studies and more experimental studies of relevant mechanisms are needed to further confirm the conclusions of this study.

## Figures and Tables

**Table 1:** Weighted baseline characteristics of the study population grouped by sex.

	Overall	Male	Female	*p*-Value
N	2015	1057	958	
Age (years, mean (SD))	47.57 (16.72)	46.09 (16.44)	49.17 (16.88)	0.007
PIR (median [IQR])	2.95 [1.49, 4.92]	3.06 [1.59, 4.92]	2.89 [1.32, 4.92]	0.121
Race, *n* (%)				0.011
Hispanic	461 (13.39)	242 (14.83)	219 (11.82)	
Non-Hispanic White	817 (69.26)	432 (70.06)	385 (68.39)	
Non-Hispanic Black	443 (10.12)	229 (8.76)	214 (11.58)	
Other races	294 (7.24)	154 (6.35)	140 (8.21)	
Education, *n* (%)				0.322
High school graduate and below	867 (37.15)	489 (38.87)	378 (35.28)	
Above high school	1148 (62.85)	568 (61.13)	580 (64.72)	
*BMI* ^1^				0.013
<25 kg/m^2^	576 (28.21)	298 (26.11)	278 (30.49)	
25–30 kg/m^2^	649 (32.50)	390 (36.92)	259 (27.71)	
≥30 kg/m^2^	790 (39.29)	369 (36.97)	421 (41.80)	
Smoker, *n* (%)				<0.001
No	1108 (55.50)	492 (48.68)	616 (62.90)	
Yes	907 (45.50)	565 (51.32)	342 (37.10)	
Cotinine, *n* (%) ^2^				0.014
<0.015 ng/mL	643 (35.01)	296 (30.60)	347 (39.78)	
≥0.015 ng/mL	1372 (64.99)	761 (69.40)	611 (60.22)	
Alcohol consumption per day (drinks, median [IQR])	0.08 [0.00, 0.57]	0.20 [0.02, 1.00]	0.03 [0.00, 0.28]	<0.001
HEI-2015 (mean (SD))	50.25 (13.86)	49.06 (13.54)	51.55 (14.10)	0.014
Physical activity, *n* (%)				<0.001
No	479 (19.40)	215 (15.39)	264 (23.73)	
Moderate	896 (45.60)	422 (39.83)	474 (51.85)	
Vigorous	640 (35.01)	420 (44.78)	220 (24.42)	
Hypertension, *n* (%)				0.190
No	1137 (61.32)	570 (59.53)	567 (63.26)	
Yes	878 (38.68)	487 (40.47)	391 (36.74)	
Diabetes, *n* (%)				0.397
No	1568 (83.11)	804 (82.12)	764 (84.18)	
Yes	447 (16.89)	253 (17.88)	194 (15.82)	
CVD, *n* (%)				0.008
No	1804 (92.09)	921 (90.31)	883 (94.01)	
Yes	211 (7.91)	136 (9.69)	75 (5.99)	
*VAI* (median [IQR])	1.33 [0.78, 2.31]	1.34 [0.76, 2.36]	1.30 [0.81, 2.25]	0.932
*LAP* (median [IQR])	42.22 [21.88, 72.13]	42.96 [23.07, 72.93]	40.95 [21.45, 69.83]	0.171

^1^ Body mass index (*BMI)* was categorized into normal weight (<25 kg/m^2^), overweight (25–30 kg/m^2^), and obesity (≥30 kg/m^2^); ^2^ serum cotinine concentration was used to determine whether an individual is tobacco-free (<0.015 ng/mL) or tobacco-exposed (≥0.015 ng/mL). Notes: PIR, poverty-to-income ratio; HEI-2015, Healthy Eating Index-2015; CVD, cardiovascular disease; SD, standard deviation; IQR, interquartile range; *VAI*, visceral adiposity index; and *LAP*, lipid accumulation product.

**Table 2:** β (95% CI) of the *VAI* and *LAP* associated with exposure to mixed mVOCs by positive and negative weighted quantile sum (WQS) regression analyses.

	Positive WQS Model		Negative WQS Model	
	β (95% CI)	*p*-value	β (95% CI)	*p*-value
*VAI*	0.084 (0.022, 0.147)	0.008	0.003 (−0.056, 0.061)	0.933
*LAP*	0.046 (−0.012, 0.105)	0.120	−0.029 (−0.090, 0.031)	0.342

## Data Availability

The original data presented in this study are openly available in the National Health and Nutrition Examination Survey at https://wwwn.cdc.gov/nchs/nhanes/Default.aspx (accessed on 7 January 2025).

## Supplementary Materials

The following supporting information can be downloaded at https://www.mdpi.com/article/10.3390/toxics13010046/s1, Text S1: Assessment of covariates; Table S1: Parent compounds, LLODs, detection rates, and descriptive statistics (*n* = 2015) for urinary mVOCs included in the analysis; Table S2: Weighted baseline characteristics of included participants divided by *VAI*-weighted tertiles; Table S3: Weighted baseline characteristics of included participants divided by *LAP*-weighted tertiles; Table S4: Weighted concentrations of mVOCs (μg/g Cr) in urine grouped by sex; Table S5: Weighted concentrations of mVOCs in urine grouped by age; Table S6: Weighted concentrations of mVOCs in urine grouped by obesity status; Table S7: Posterior inclusion probability (PIP) of VOC metabolites derived from BKMR modeling; Figure S1: Flowchart of the participant screening process for inclusion in the analysis; Figure S2: RCS results of the relationships between urinary mVOCs and the *VAI*; Figure S3: RCS results of the relationships between urinary mVOCs and the *LAP*.

## Abbreviations

WHO, World Health Organization; *BMI*, body mass index; VOCs, volatile organic compounds; mVOCs, volatile organic compound metabolites; *WC*, waist circumference; *VAI*, visceral adiposity index; *LAP*, lipid accumulation product; NHANES, National Health and Nutrition Examination Survey; UPLC-ESI/MSMS, ultra-performance liquid chromatography–electrospray tandem mass spectrometry; LLODs, lower limits of detection; CEMA, N-acetyl-S-(2-carboxyethyl)-L-cysteine; HMPMA, N-acetyl-S-(3-hydroxypropyl-1-methyl)-L-cysteine; MHBMA3, N-acetyl-S-(4-hydroxy-2-butenyl)-L-cysteine; CYMA, N-acetyl-S-(2-cyanoethyl)-L-cysteine; AMCC, N-acetyl-S-(N-methylcarbamoyl)-L-cysteine; 2HPMA, N-acetyl-S-(2-hydroxypropyl)-L-cysteine; 2MHA, 2-methylhippuric acid; 3HPMA, N-acetyl-S-(3-hydroxypropyl)-L-cysteine; 3-4MHA, 3- and 4-methylhippuric acid; AAMA, N-acetyl-S-(2-carbamoylethyl)-L-cysteine; ATCA, 2-aminothiazoline-4-carboxylic acid; BPMA, N-acetyl-S-(n-propyl)-L-cysteine; DHBMA, N-acetyl-S-(3,4-dihydroxybutyl)-L-cysteine; MA, mandelic acid; BMA, N-acetyl-S-(benzyl)-L-cysteine; PGA, phenylglyoxylic acid; *TGs*, triglycerides; *HDL-C*, high-density lipoprotein cholesterol; PIR, poverty-to-income ratio; HEI-2015, Healthy Eating Index-2015; CVD, cardiovascular disease; SD, standard deviation; IQR, interquartile range; WGLM, survey-weighted generalized linear models; RCS, restricted cubic spline; WQS, weighted quantile sum; BKMR, Bayesian kernel machine regression; ROS, reactive oxygen species.
