# Supplementary material for: Exposure to Volatile Organic Compounds in Relation to Visceral Adiposity Index and Lipid Accumulation Product Among U.S. Adults: NHANES 2011–2018
Source: Toxics. 2025 Jan 9;13(1):46. doi: 10.3390/toxics13010046 (PMC11768580; doi:10.3390/toxics13010046)
Supplement: Supplementary file 1 [file toxics-13-00046-s001.zip › toxics-3322442-supplementary.pdf]

*Supplementary materials*

# Exposure to Volatile Organic Compounds in Relation to Visceral Adiposity Index and Lipid Accumulation Product among Adults: NHANES 2011-2018

**Ziyi Qian [1], Chenxu Dai [1], Siyan Chen [1], Linjie Yang [1,2] and Xia Huo [1,*]**

[1] Laboratory of Environmental Medicine and Developmental Toxicology, Guangdong Key Laboratory of Environmental Pollution and Health, College of Environment and Climate, Jinan University, Guangzhou 511443, China
[2] Department of Public Health and Preventive Medicine, School of Medicine, Jinan University, Guangzhou 510632, China
* Correspondence: xhuo@jnu.edu.cn

**Text S1.** Assessment of covariates;

**Table S1.** Parent compounds, LLODs, detection rates, and descriptive statistics (n = 2015) for urinary mVOCs included in the analysis;

**Table S2.** Weighted baseline characteristics of included participants divided by VAI-weighted tertiles;

**Table S3.** Weighted baseline characteristics of included participants divided by LAP-weighted tertiles;

**Table S4.** Weighted median [IQR] concentrations of mVOCs (μg/g Cr) in urine grouped by sex;

**Table S5.** Weighted median [IQR] concentrations of mVOCs in urine grouped by age;

**Table S6.** Weighted median [IQR] concentrations of mVOCs in urine grouped by obesity status;

**Table S7.** Posterior inclusion probability (PIP) of VOC metabolites derived from BKMR modeling;

**Figure S1.** Flowchart of the participant screening process for inclusion in the analysis;

**Figure S2.** RCS results of the relationships between urinary mVOCs and VAI;

**Figure S3.** RCS results of the relationships between urinary mVOCs and LAP.

## Text S1. Assessment of covariates

The DEMO module of the NHANES database provides demographic information, including gender (male or female), age (continuous), race (Hispanic, non-Hispanic White, non-Hispanic Black, and other races), education level (high school graduate and below, and above high school), and PIR (continuous). BMI was categorized into three levels: normal weight (< 25 $kg/m^2$), overweight (25–30 $kg/m^2$), and obesity (≥ 30 $kg/m^2$). Having smoked at least 100 cigarettes in a lifetime was defined as a self-reported smoker. Serum cotinine is a biomarker of nicotine exposure with a lower limit of detection of 0.015 ng/mL. Serum cotinine concentration was used to determine whether an individual is tobacco-free (< 0.015 ng/mL) or tobacco-exposed (≥ 0.015 ng/mL). Average daily alcohol consumption in the past 12 months (continuous) was calculated based on the alcohol use data [1]. Different types of physical activity have different metabolic equivalent (MET) values. Physical activity was calculated based on the type of activity, MET value, weekly frequency, and duration. Physical activity was finally categorized into three levels: no physical activity (< 1 MET-h/wk), moderate physical activity (1-48 MET-h/wk), and vigorous physical activity (> 48 MET-h/wk) [2]. HEI-2015 is an indicator that assesses whether a set of foods aligns with the 2015-2020 Dietary Guidelines for Americans, with a maximum score of 100, and a higher score indicates a healthier diet [3]. The HEI-2015 (continuous) in this study was assessed based on data from the first 24 hour dietary recall interview. Participants were considered to have diabetes if they met one of the following conditions: fasting blood glucose ≥ 126 mg/dL, HbA1c level ≥ 6.5%, oral glucose tolerance test ≥ 200 mg/dL, a self-reported medical diagnosis, or current use of oral hypoglycemic medication or insulin [4]. Hypertension was defined as a systolic blood pressure ≥ 140 mmHg or a diastolic blood pressure ≥ 90 mmHg, or receiving a medical diagnosis from a healthcare professional [5]. Self-reported medical diagnoses of congestive heart failure, coronary heart disease, angina pectoris, heart attack, or stroke were defined as CVD [6].

**Table S1. Parent compounds, LLODs, detection rates, and descriptive statistics (*n* = 2015) for urinary mVOCs included in the analysis.**

| VOC metabolites | Abbreviation | Parent compound | LLOD (μg/L) | Detection rate (%) | Median [IQR] (ng/mL） |
|---|---|---|---|---|---|
| 2-methylhippuric acid | 2MHA | Xylene | 5 | 92.36 | 33.80 [13.35, 82.60] |
| 3- and 4-methylhippuric acid | 3-4MHA | Xylene | 8 | 99.60 | 230.00 [93.40, 600.50] |
| N-acetyl-S-(2-carbamoylethyl)-L-cysteine | AAMA | Acrylamide | 2.2 | 99.95 | 53.80 [28.75, 108.00] |
| N-acetyl-S-(N-methylcarbamoyl)-L-cysteine | AMCC | N, N-Dimethylformamide | 6.26 | 99.90 | 166.00 [85.45, 323.00] |
| 2-aminothiazoline-4-carboxylic acid | ATCA | Cyanide | 15 | 93.45 | 105.00 [50.65, 205.00] |
| N-acetyl-S-(benzyl)-L-cysteine | BMA | Toluene | 0.5 | 99.80 | 6.75 [3.66, 13.30] |
| N-acetyl-S-(n-propyl)-L-cysteine | BPMA | 1-Bromopropane | 1.2 | 79.55 | 4.13 [1.51, 10.95] |
| N-acetyl-S-(2-carboxyethyl)-L-cysteine | CEMA | Acrolein | 6.96 | 99.55 | 100.00 [53.50, 189.00] |
| N-acetyl-S-(2-cyanoethyl)-L-cysteine | CYMA | Acrylonitrile | 0.5 | 87.84 | 1.71 [0.87, 14.60] |
| N-acetyl-S-(3,4-dihydroxybutyl)-L-cysteine | DHBMA | 1,3-Butadiene | 5.25 | 100.00 | 325.00 [197.00, 501.50] |
| N-acetyl-S-(2-hydroxypropyl)-L-cysteine | 2HPMA | Propylene oxide | 5.3 | 97.22 | 32.40 [17.30, 62.20] |
| N-acetyl-S-(3-hydroxypropyl)-L-cysteine | 3HPMA | Acrolein | 13 | 99.90 | 224.00 [121.50, 444.50] |
| Mandelic acid | MA | Styrene | 12 | 99.06 | 136.00 [80.15, 232.00] |
| N-acetyl-S-(4-hydroxy-2-butenyl)-L-cysteine | MHBMA3 | 1,3-Butadiene | 0.6 | 97.22 | 4.47 [2.38, 9.22] |
| Phenylglyoxylic acid | PGA | Ethylbenzene, styrene | 12 | 99.90 | 226.00 [135.00, 364.00] |
| N-acetyl-S-(3-hydroxypropyl-1-methyl)-L-cysteine | HMPMA | Crotonaldehyde | 1.7 | 100.00 | 214.00 [123.00, 388.00] |

**Table S2. Weighted baseline characteristics of included participants divided by VAI-weighted tertiles.**

| | Tertile 1 | Tertile 2 | Tertile 3 | *p*-Value |
|---|---|---|---|---|
| **N** | 663 | 686 | 666 | |
| **Sex, *n* (%)** | | | | 0.173 |
| Male | 361 (53.85) | 355 (48.03) | 341 (54.25) | |
| Female | 302 (46.15) | 331 (51.97) | 325 (45.75) | |
| **Age (years, mean (SD))** | 44.99 (18.00) | 48.35 (16.29) | 49.35 (15.51) | 0.001 |
| **PIR (median [IQR])** | 3.23 [1.76, 5.00] | 3.04 [1.42, 4.74] | 2.56 [1.31, 4.62] | 0.003 |
| **Race, *n* (%)** | | | | 0.001 |
| Hispanic | 99 (9.72) | 179 (15.21) | 183 (15.18) | |
| Non-Hispanic White | 245 (68.54) | 274 (68.94) | 298 (70.29) | |
| Non-Hispanic Black | 204 (14.14) | 141 (9.13) | 98 (7.10) | |
| Other race | 115 (7.60) | 92 (6.71) | 87 (7.43) | |
| **Education, *n* (%)** | | | | 0.008 |
| High school graduate and below | 238 (30.95) | 300 (37.53) | 329 (42.96) | |
| Above high school | 425 (69.05) | 386 (62.47) | 337 (57.04) | |
| **BMI, *n* (%)** | | | | < 0.001 |
| < 25 kg/m$^2$ | 321 (50.11) | 160 (22.36) | 95 (12.33) | |
| 25-30 kg/m$^2$ | 194 (27.91) | 254 (37.77) | 201 (31.67) | |
| ≥ 30 kg/m$^2$ | 148 (21.98) | 272 (39.87) | 370 (56.01) | |
| **Smoker, *n* (%)** | | | | 0.111 |
| No | 397 (60.23) | 371 (54.83) | 340 (51.46) | |
| Yes | 266 (39.77) | 315 (45.17) | 326 (48.54) | |
| **Cotinine, *n* (%)** | | | | 0.760 |
| < 0.015 ng/mL | 206 (36.21) | 234 (35.48) | 203 (33.31) | |
| ≥ 0.015 ng/mL | 457 (63.79) | 452 (64.52) | 463 (66.69) | |
| **Alcohol consumption per day (drinks, median [IQR])** | 0.16 [0.01, 0.86] | 0.07 [0.00, 0.44] | 0.07 [0.00, 0.49] | 0.002 |
| **HEI-2015 (mean (SD))** | 52.47 (14.31) | 49.29 (13.71) | 49.01 (13.30) | 0.004 |
| **Physical activity, *n* (%)** | | | | < 0.001 |
| No | 116 (14.71) | 160 (18.24) | 203 (25.28) | |
| Moderate | 290 (40.30) | 311 (48.53) | 295 (47.87) | |
| Vigorous | 257 (44.99) | 215 (33.23) | 168 (26.85) | |
| **Hypertension, *n* (%)** | | | | < 0.001 |
| No | 430 (73.14) | 375 (56.76) | 332 (54.20) | |
| Yes | 233 (26.86) | 311 (43.24) | 334 (45.80) | |

| | | | | |
|---|---|---|---|---|
| **Diabetes, *n* (%)** | | | | < 0.001 |
| No | 589 (93.99) | 555 (83.81) | 424 (71.50) | |
| Yes | 74 (6.01) | 131 (16.19) | 242 (28.50) | |
| **CVD, *n* (%)** | | | | 0.001 |
| No | 617 (94.77) | 619 (93.50) | 568 (87.93) | |
| Yes | 46 (5.23) | 67 (6.50) | 98 (12.07) | |
| **2MHA** | 26.64 [13.26, 75.45] | 33.05 [12.52, 81.03] | 36.64 [13.66, 84.70] | 0.298 |
| **3-4MHA** | 210.52 [79.43, 564.15] | 250.83 [90.68, 616.65] | 254.10 [97.25, 580.29] | 0.136 |
| **AAMA** | 57.83 [36.71, 102.69] | 49.46 [31.68, 99.62] | 51.46 [34.25, 97.03] | 0.221 |
| **AMCC** | 164.10 [87.72, 244.43] | 167.47 [102.33, 292.52] | 183.78 [111.15, 354.51] | 0.010 |
| **ATCA** | 99.16 [53.34, 188.85] | 104.42 [56.44, 200.73] | 103.69 [54.17, 196.11] | 0.674 |
| **BMA** | 6.88 [4.24, 13.30] | 6.39 [3.87, 10.50] | 6.17 [3.93, 10.56] | 0.094 |
| **BPMA** | 5.14 [2.12, 11.01] | 3.82 [1.73, 10.25] | 4.04 [1.55, 10.33] | 0.188 |
| **CEMA** | 80.86 [53.36, 137.35] | 92.07 [57.29, 147.91] | 99.95 [66.02, 181.84] | < 0.001 |
| **CYMA** | 1.46 [0.92, 3.43] | 1.42 [0.85, 8.33] | 1.66 [0.88, 45.26] | 0.028 |
| **DHBMA** | 303.84 [242.20, 400.15] | 305.13 [237.25, 388.00] | 325.04 [248.33, 412.81] | 0.193 |
| **2HPMA** | 30.56 [19.00, 52.72] | 29.38 [19.48, 51.89] | 30.19 [18.28, 55.22] | 0.776 |
| **3HPMA** | 195.76 [124.01, 328.30] | 183.82 [125.48, 318.60] | 219.06 [131.37, 422.29] | 0.036 |
| **MA** | 127.97 [97.17, 181.00] | 123.96 [90.02, 178.60] | 134.20 [94.75, 200.04] | 0.069 |
| **MHBMA3** | 3.60 [2.51, 6.19] | 3.43 [2.41, 5.76] | 4.11 [2.70, 10.26] | < 0.001 |
| **PGA** | 217.41 [165.56, 289.98] | 215.36 [158.76, 296.24] | 222.87 [156.62, 321.94] | 0.566 |
| **HMPMA** | 172.28 [141.74, 238.81] | 175.29 [136.76, 254.45] | 196.53 [145.37, 363.05] | 0.001 |

**Table S3. Weighted baseline characteristics of included participants divided by LAP-weighted tertiles**

| | **Tertile 1** | **Tertile 2** | **Tertile 3** | ***p*-Value** |
|---|---|---|---|---|
| **N** | 647 | 725 | 643 | |
| **Sex, *n* (%)** | | | | 0.292 |
| Male | 337 (49.13) | 375 (51.50) | 345 (55.39) | |
| Female | 310 (50.87) | 350 (48.50) | 298 (44.61) | |
| **Age (years, mean (SD))** | 43.00 (17.46) | 49.21 (16.81) | 50.44 (14.84) | < 0.001 |
| **PIR (median [IQR])** | 3.15 [1.74, 5.00] | 3.11 [1.46, 5.00] | 2.58 [1.33, 4.37] | 0.003 |
| **Race, *n* (%)** | | | | 0.002 |
| Hispanic | 105 (10.29) | 174 (14.40) | 182 (15.43) | |
| Non-Hispanic White | 249 (69.77) | 268 (66.07) | 300 (72.03) | |
| Non-Hispanic Black | 167 (11.87) | 179 (11.69) | 97 (6.74) | |
| Other race | 126 (8.07) | 104 (7.84) | 64 (5.79) | |

| | | | | |
|---|---|---|---|---|
| **Education, *n* (%)** | | | | 0.009 |
| High school graduate and below | 231 (31.81) | 320 (35.81) | 316 (43.86) | |
| Above high school | 416 (68.19) | 405 (64.19) | 327 (56.14) | |
| **BMI, *n* (%)** | | | | < 0.001 |
| < 25 kg/m$^2$ | 408 (64.40) | 140 (17.46) | 28 (3.17) | |
| 25-30 kg/m$^2$ | 183 (27.26) | 306 (44.18) | 160 (25.68) | |
| ≥ 30 kg/m$^2$ | 56 (8.35) | 279 (38.36) | 455 (71.15) | |
| **Smoker, *n* (%)** | | | | 0.096 |
| No | 394 (60.66) | 393 (54.23) | 321 (51.68) | |
| Yes | 253 (39.34) | 332 (45.77) | 322 (48.32) | |
| **Cotinine, *n* (%)** | | | | 0.681 |
| < 0.015 ng/mL | 188 (33.63) | 247 (36.65) | 208 (34.68) | |
| ≥ 0.015 ng/mL | 459 (66.37) | 478 (63.35) | 435 (65.32) | |
| **Alcohol consumption per day (drinks, median [IQR])** | 0.14 [0.01, 0.71] | 0.08 [0.00, 0.57] | 0.05 [0.00, 0.43] | 0.007 |
| **HEI-2015 (mean (SD))** | 52.13 (14.43) | 51.03 (13.63) | 47.57 (13.13) | < 0.001 |
| **Physical activity, *n* (%)** | | | | 0.001 |
| No | 105 (14.45) | 184 (20.62) | 190 (23.07) | |
| Moderate | 291 (41.87) | 317 (45014) | 288 (49.78) | |
| Vigorous | 251 (43.68) | 224 (34.24) | 165 (27.15) | |
| **Hypertension, *n* (%)** | | | | < 0.001 |
| No | 466 (77.22) | 391 (61.69) | 280 (45.06) | |
| Yes | 181 (22.78) | 334 (38.31) | 363 (54.94) | |
| **Diabetes, *n* (%)** | | | | < 0.001 |
| No | 598 (95.51) | 573 (85.94) | 397 (67.82) | |
| Yes | 49 (4.49) | 152 (14.06) | 246 (32.18) | |
| **CVD, *n* (%)** | | | | 0.008 |
| No | 602 (94.87) | 649 (92.46) | 553 (88.92) | |
| Yes | 45 (5.13) | 76 (7.54) | 90 (11.08) | |
| **2MHA** | 28.00 [13.43, 79.90] | 39.16 [13.11, 88.21] | 31.25 [12.56, 77.44] | 0.357 |
| **3-4MHA** | 222.72 [80.31, 616.57] | 253.89 [83.19, 616.21] | 193.58 [94.00, 528.29] | 0.658 |
| **AAMA** | 59.19 [36.43, 110.19] | 51.14 [33.15, 92.76] | 49.57 [33.31, 92.19] | 0.053 |
| **AMCC** | 163.22 [81.31, 272.18] | 175.20 [106.03, 293.17] | 176.52 [112.55, 330.08] | 0.025 |
| **ATCA** | 100.69 [57.03, 184.59] | 105.59 [56.70, 211.88] | 101.74 [50.47, 183.08] | 0.210 |
| **BMA** | 7.04 [4.27, 12.82] | 6.37 [3.84, 11.41] | 5.90 [3.99, 10.52] | 0.059 |
| **BPMA** | 5.57 [2.23, 12.15] | 3.86 [1.76, 9.23] | 3.82 [1.47, 9.50] | 0.009 |
| **CEMA** | 77.73 [51.63, 140.71] | 95.11 [57.77, 150.89] | 98.63 [67.27, 170.31] | < 0.001 |
| **CYMA** | 1.49 [0.94, 6.74] | 1.42 [0.85, 4.40] | 1.56 [0.86, 28.31] | 0.385 |

| | | | | |
|---|---|---|---|---|
| **DHBMA** | 301.12 [238.76, 396.54] | 306.68 [242.06, 395.38] | 327.45 [249.90, 416.17] | 0.098 |
| **2HPMA** | 32.74 [21.10, 57.44] | 30.62 [17.79, 52.48] | 27.28 [18.22, 52.31] | 0.037 |
| **3HPMA** | 199.10 [123.38, 368.50] | 185.63 [124.62, 319.93] | 205.64 [131.68, 343.78] | 0.359 |
| **MA** | 127.41 [91.80, 185.08] | 128.91 [95.08, 180.94] | 128.67 [92.17, 188.51] | 0.972 |
| **MHBMA3** | 3.57 [2.46, 6.65] | 3.62 [2.52, 6.22] | 3.91 [2.61, 8.63] | 0.245 |
| **PGA** | 218.78 [166.80, 291.81] | 219.54 [165.81, 303.67] | 212.93 [153.87, 310.56] | 0.545 |
| **HMPMA** | 172.54 [140.44, 249.43] | 173.17 [135.83, 266.20] | 196.52 [146.08, 338.54] | 0.008 |

**Table S4. Weighted median [IQR] concentrations of mVOCs (μg/g Cr) in urine grouped by sex.**

| mVOCs | Total | Male (*n* = 1057) | Female (*n* = 958) | *p*-Value |
|---|---|---|---|---|
| 2MHA | 32.42 [13.20, 79.90] | 31.22 [13.53, 69.86] | 34.22 [12.55, 94.59] | 0.161 |
| 3-4MHA | 232.13 [84.16, 591.00] | 217.08 [80.88, 510.56] | 256.77 [87.98, 714.06] | 0.019 |
| AAMA | 53.24 [33.87, 100.25] | 52.41 [32.79, 101.85] | 53.57 [34.72, 94.78] | 0.768 |
| AMCC | 169.05 [102.33, 301.13] | 156.49 [91.51, 249.13] | 188.13 [116.72, 352.14] | < 0.001 |
| ATCA | 103.26 [54.88, 194.64] | 66.50 [38.46, 116.10] | 171.31 [94.60, 258.61] | < 0.001 |
| BMA | 6.45 [4.04, 11.26] | 5.58 [3.54, 9.22] | 7.41 [4.83, 13.75] | < 0.001 |
| BPMA | 4.47 [1.76, 10.48] | 4.72 [1.74, 10.35] | 4.25 [1.77, 10.62] | 0.963 |
| CEMA | 92.62 [56.99, 153.69] | 90.75 [57.41, 149.38] | 93.49 [56.67, 156.30] | 0.858 |
| CYMA | 1.48 [0.88, 9.67] | 1.46 [0.86, 17.02] | 1.53 [0.91, 4.43] | 0.932 |
| DHBMA | 314.43 [243.02, 403.76] | 294.11 [232.73, 393.86] | 325.00 [254.50, 412.87] | 0.001 |
| 2HPMA | 30.14 [18.99, 53.15] | 29.74 [19.00, 51.89] | 30.54 [18.90, 56.37] | 0.854 |
| 3HPMA | 197.52 [127.36, 348.79] | 209.18 [141.63, 371.25] | 174.61 [112.43, 302.84] | < 0.001 |
| MA | 128.65 [93.48, 185.18] | 119.13 [87.56, 173.27] | 138.74 [98.33, 195.32] | 0.002 |
| MHBMA3 | 3.67 [2.55, 6.90] | 3.71 [2.56, 7.30] | 3.67 [2.55, 6.23] | 0.885 |
| PGA | 217.96 [162.27, 303.17] | 199.81 [151.00, 270.59] | 241.30 [179.18, 336.40] | < 0.001 |
| HMPMA | 179.82 [140.70, 274.60] | 175.39 [136.30, 284.67] | 183.29 [145.27, 274.34] | 0.149 |

**Table S5. Weighted median [IQR] concentrations of mVOCs in urine grouped by age.**

| mVOCs | < 60 years (*n* = 1338) | ≥ 60 years (*n* = 677) | *p*-Value |
|---|---|---|---|
| 2MHA | 32.95 [13.43, 82.42] | 28.65 [11.94, 75.50] | 0.234 |
| 3-4MHA | 242.76 [80.97, 588.44] | 221.01 [91.90, 607.74] | 0.710 |
| AAMA | 55.96 [34.35, 106.76] | 48.97 [32.90, 80.10] | 0.023 |
| AMCC | 165.82 [95.76, 292.67] | 181.89 [119.29, 310.77] | 0.050 |
| ATCA | 100.00 [53.50, 185.24] | 118.97 [58.74, 220.50] | 0.081 |

| | | | |
|---|---|---|---|
| BMA | 6.19 [3.85, 10.60] | 6.95 [4.40, 12.90] | 0.011 |
| BPMA | 4.61 [1.77, 10.48] | 4.13 [1.73, 10.44] | 0.539 |
| CEMA | 87.44 [56.04, 152.59] | 99.00 [63.05, 162.14] | 0.008 |
| CYMA | 1.62 [0.89, 33.42] | 1.23 [0.85, 2.08] | < 0.001 |
| DHBMA | 293.80 [231.53, 378.09] | 372.84 [277.42, 448.11] | < 0.001 |
| 2HPMA | 29.78 [18.98, 52.74] | 30.89 [18.97, 56.29] | 0.519 |
| 3HPMA | 202.89 [128.19, 379.53] | 186.50 [124.14, 293.99] | 0.025 |
| MA | 126.64 [93.08, 187.45] | 132.04 [93.94, 178.07] | 0.907 |
| MHBMA3 | 3.54 [2.40, 8.09] | 3.99 [2.98, 5.69] | 0.087 |
| PGA | 206.48 [157.10, 296.10] | 238.13 [185.15, 306.02] | 0.002 |
| HMPMA | 170.48 [132.23, 274.46] | 200.74 [161.12, 280.06] | < 0.001 |

**Table S6. Weighted median [IQR] concentrations of mVOCs in urine grouped by obesity status.**

| mVOCs | Non-obesity (*n* = 1225) | Obesity (*n* = 790) | *p*-Value |
|---|---|---|---|
| 2MHA | 37.49 [14.11, 93.29] | 26.36 [11.72, 64.01] | < 0.001 |
| 3-4MHA | 285.90 [88.40, 672.26] | 167.95 [80.55, 494.71] | < 0.001 |
| AAMA | 57.86 [34.07, 107.01] | 48.73 [33.59, 85.43] | 0.007 |
| AMCC | 174.73 [100.29, 306.04] | 163.77 [104.08, 290.48] | 0.518 |
| ATCA | 103.42 [55.63, 194.36] | 103.12 [52.52, 195.57] | 0.972 |
| BMA | 6.97 [4.20, 12.73] | 5.75 [3.75, 9.50] | 0.003 |
| BPMA | 4.95 [1.93, 10.79] | 3.88 [1.59, 9.54] | 0.050 |
| CEMA | 87.59 [54.86, 150.91] | 97.19 [65.74, 156.84] | 0.015 |
| CYMA | 1.54 [0.93, 12.93] | 1.41 [0.82, 7.58] | 0.020 |
| DHBMA | 315.88 [244.75, 403.73] | 306.46 [242.43, 402.61] | 0.703 |
| 2HPMA | 32.55 [20.17, 58.41] | 26.84 [17.69, 47.62] | 0.005 |
| 3HPMA | 196.74 [125.84, 376.76] | 201.51 [130.85, 308.40] | 0.469 |
| MA | 131.08 [95.10, 190.80] | 120.91 [89.76, 178.12] | 0.032 |
| MHBMA3 | 3.74 [2.56, 7.03] | 3.54 [2.53, 6.41] | 0.358 |
| PGA | 226.53 [166.12, 306.13] | 202.60 [154.70, 296.03] | 0.011 |
| HMPMA | 180.00 [139.60, 282.40] | 179.63 [142.43, 269.35] | 0.618 |

Notes: The obesity status of participants was determined by their BMI, with a BMI ≥ 30 kg/$m^2$ identified as obesity.

**Table S7. Posterior inclusion probability (PIP) of VOC metabolites derived from BKMR modeling.**

| Variable | VAI | LAP |
|---|---|---|
| 2MHA | 0.1838 | 0 |
| 3-4MHA | 0.2120 | 0.0336 |
| AAMA | 0.6414 | 0.2040 |
| AMCC | 0.9914 | 0.8938 |
| ATCA | 0.4250 | 0 |
| BMA | 0.2066 | 0 |
| BPMA | 0.1072 | 0 |
| CEMA | 0.0904 | 0.0114 |
| CYMA | 0.4770 | 0.4698 |
| DHBMA | 0.0876 | 0 |
| 2HPMA | 0.2714 | 0.1018 |
| 3HPMA | 0.2758 | 0 |
| MA | 0.2724 | 0 |
| MHBMA3 | 0.5330 | 0.0124 |
| PGA | 0.9128 | 0.1234 |
| HMPMA | 0.5058 | 0.0028 |

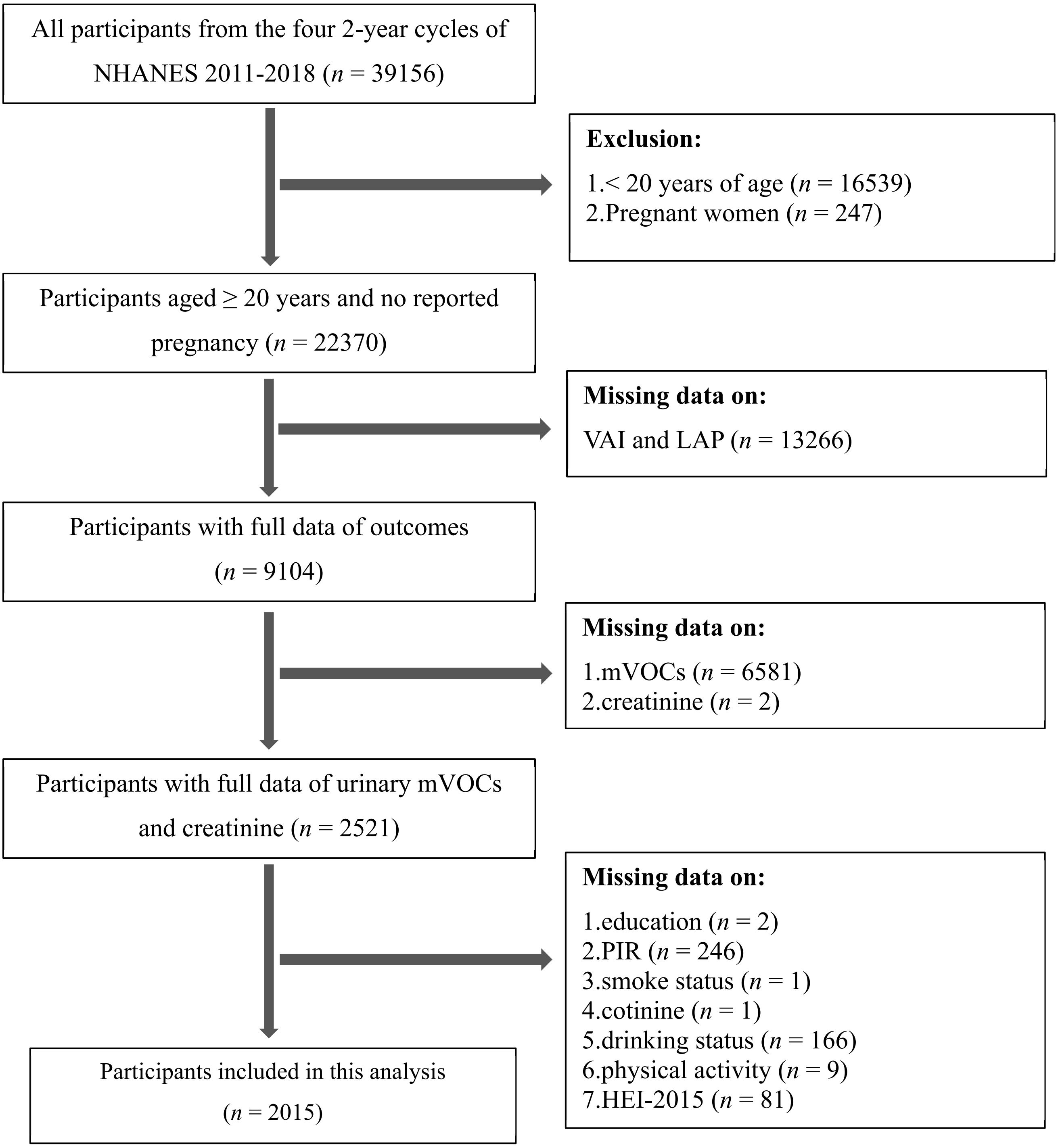


**Figure S1. Flowchart of the participant screening process for inclusion in the analysis.**

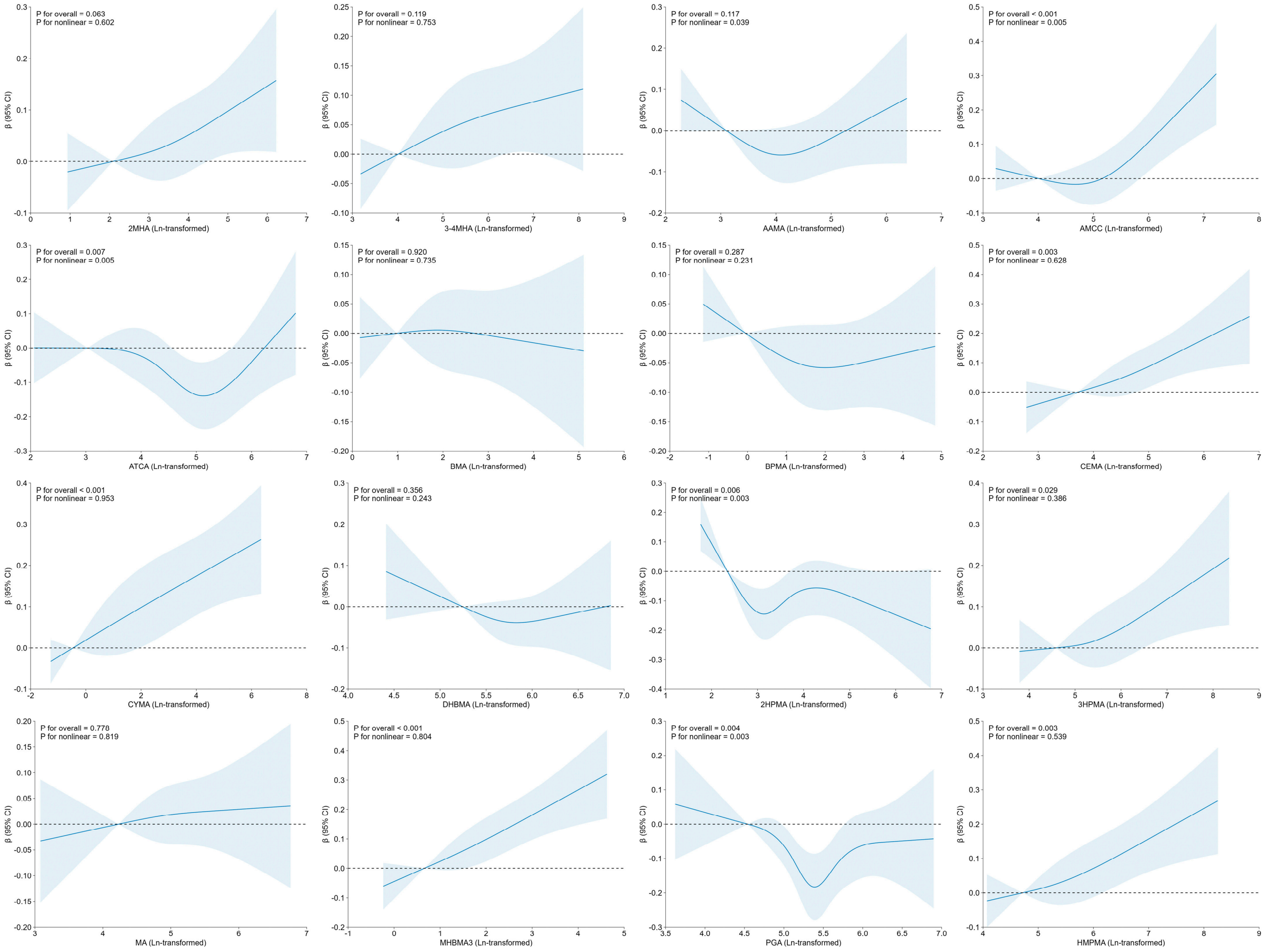


**Figure S2. RCS results of the relationships between urinary mVOCs and VAI. The models were adjusted by sex, age, race, education, PIR, BMI, physical activity, HEI-2015, self-reported smoking status, average daily alcohol consumption, diabetes, hypertension, and CVD.**

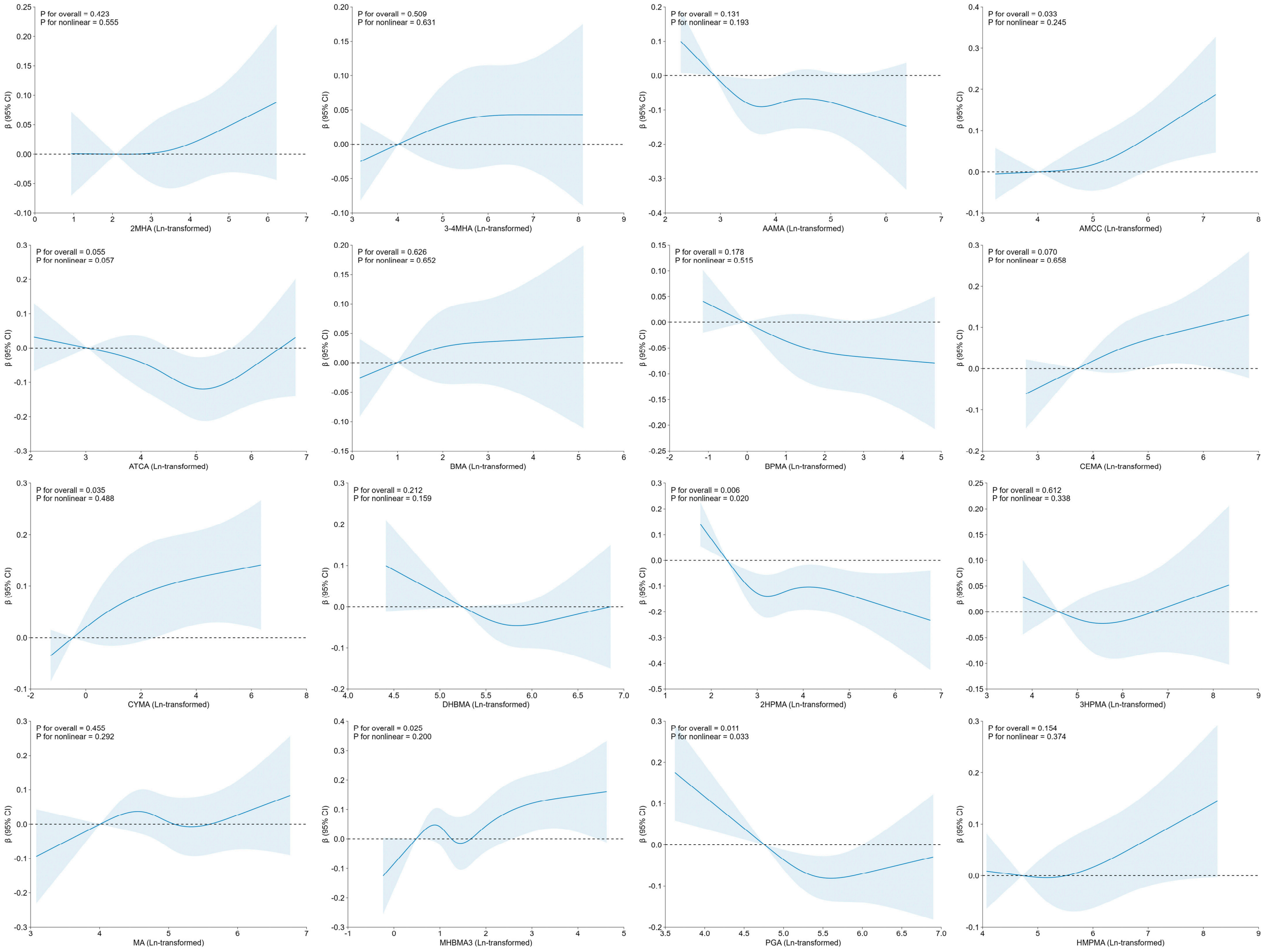


**Figure S3. RCS results of the relationships between urinary mVOCs and LAP. The models were adjusted by sex, age, race, education, PIR, BMI, physical activity, HEI-2015, self-reported smoking status, average daily alcohol consumption, diabetes, hypertension, and CVD.**

# Reference


1. Tan, Y.; Fu, Y.; Yao, H.; Li, H.; Wu, X.; Guo, Z.; Liang, X.; Kuang, M.; Tan, L.; Jing, C. The relationship of organophosphate flame retardants with hyperuricemia and gout via the inflammatory response: An integrated approach. *Sci Total Environ* **2024**, *908*, 168169, doi:10.1016/j.scitotenv.2023.168169.
2. Tian, X.; Xue, B.; Wang, B.; Lei, R.; Shan, X.; Niu, J.; Luo, B. Physical activity reduces the role of blood cadmium on depression: A cross-sectional analysis with NHANES data. *Environ Pollut* **2022**, *304*, 119211, doi:10.1016/j.envpol.2022.119211.
3. Krebs-Smith, S.M.; Pannucci, T.E.; Subar, A.F.; Kirkpatrick, S.I.; Lerman, J.L.; Tooze, J.A.; Wilson, M.M.; Reedy, J. Update of the Healthy Eating Index: HEI-2015. *J Acad Nutr Diet* **2018**, *118*, 1591-1602, doi:10.1016/j.jand.2018.05.021.
4. Wang, X.; He, W.; Wu, X.; Song, X.; Yang, X.; Zhang, G.; Niu, P.; Chen, T. Exposure to volatile organic compounds is a risk factor for diabetes: A cross-sectional study. *Chemosphere* **2023**, *338*, 139424, doi:10.1016/j.chemosphere.2023.139424.
5. Lu, L.; Ni, R. Association between polycyclic aromatic hydrocarbon exposure and hypertension among the U.S. adults in the NHANES 2003-2016: A cross-sectional study. *Environ Res* **2023**, *217*, 114907, doi:10.1016/j.envres.2022.114907.

6. Wang, X.; Chen, Z.; Cheng, D.; Cao, Y.; Xie, X.; Zhou, J.; Wu, Y.; Li, X.; Yu, J.; Yang, B. Association between urinary metabolites of volatile organic compounds and cardiovascular disease in the general population from NHANES 2011-2018. *Ecotoxicol Environ Saf* **2023**, *264*, 115412, doi:10.1016/j.ecoenv.2023.115412.
